# Prosthetic Valve Endocarditis After Aortic Valve Replacement With Bovine Versus Porcine Bioprostheses

**DOI:** 10.1161/JAHA.123.031387

**Published:** 2023-12-29

**Authors:** Natalie Glaser, Ulrik Sartipy, Michael Dismorr

**Affiliations:** ^1^ Department of Cardiology Stockholm South General Hospital Stockholm Sweden; ^2^ Department of Molecular Medicine and Surgery Karolinska Institutet Stockholm Sweden; ^3^ Department of Cardiothoracic Surgery Karolinska University Hospital Stockholm Sweden

**Keywords:** aortic bioprostheses, endocarditis, regression standardization, surgical aortic valve replacement, xenograft, Aortic Valve Replacement/Transcather Aortic Valve Implantation

## Abstract

**Background:**

Whether a bovine or porcine aortic valve bioprosthesis carries a higher risk of endocarditis after aortic valve replacement is unknown. The aim of this study was to compare the risk of prosthetic endocarditis in patients undergoing aortic valve replacement with a bovine versus porcine bioprosthesis.

**Methods and Results:**

This nationwide, population‐based cohort study included all patients who underwent surgical aortic valve replacement with a bovine or porcine bioprosthesis in Sweden from 1997 to 2018. Regression standardization was used to account for intergroup differences. The primary outcome was prosthetic valve endocarditis, and the secondary outcomes were all‐cause mortality and early prosthetic valve endocarditis. During a maximum follow‐up time of 22 years, we included 21 022 patients, 16 603 with a bovine valve prosthesis and 4419 with a porcine valve prosthesis. The mean age was 73 years, and 61% of the patients were men. In total, 910 patients were hospitalized for infective endocarditis: 690 (4.2%) in the bovine group and 220 (5.0%) in the porcine group. The adjusted cumulative incidence of prosthetic valve endocarditis at 15 years was 9.5% (95% CI, 6.2%–14.4%) in the bovine group and 2.8% (95% CI, 1.4%–5.6%) in the porcine group. The absolute risk difference between the groups at 15 years was 6.7% (95% CI, 0.8%–12.5%).

**Conclusions:**

The risk of endocarditis was higher in patients who received a bovine compared with a porcine valve prosthesis after surgical aortic valve replacement. This association should be considered in patients undergoing both surgical and transcatheter aortic valve replacement.

**Nonstandard Abbreviations and Acronyms**
PVE: prosthetic valve endocarditis
SAVR: surgical aortic valve replacement
SWEDEHEART: Swedish Web System for Enhancement and Development of Evidence‐Based Care in Heart Disease Evaluated According to Recommended Therapies
TAVR: transcatheter aortic valve replacement

**Clinical Perspective**

**What Is New?**

- Bovine xenograft material in bioprosthetic aortic valves is associated with a higher risk for prosthetic valve endocarditis compared with porcine xenograft material.
- The use of regression standardization helps to isolate the estimated effect of xenograft material from the effect of valve model on the outcomes.

**What Are the Clinical Implications?**

- The association between bovine graft material and prosthetic valve endocarditis should be considered when treating patients with bioprosthetic aortic valves.
- The mechanism behind this difference should be investigated and considered when developing new bioprosthetic valve models to optimize patient prognosis.

Infective endocarditis is a severe disease with an in‐hospital mortality rate of 10% to 30%, even with treatment.^1^, ^2^ Risk factors for endocarditis include intravenous drug use, older age, congenital heart defects, and prosthetic heart valves.^3^ Prosthetic valve endocarditis (PVE) is a severe form of endocarditis that can lead to valve dysfunction, heart failure, embolic stroke, and death. It accounts for ≈20% of all cases of infective endocarditis.^4^, ^5^ Patients with prosthetic aortic valves carry an ≈0.5% yearly risk of endocarditis.^6^ Prior research found that PVE may be more common in patients with biological than mechanical aortic valves.^6^, ^7^ Nevertheless, the use of biological valve prostheses in patients who undergo surgical aortic valve replacement (SAVR) has increased during the past decade,^8^ and in patients who undergo transcatheter aortic valve replacement (TAVR), biological valve prostheses are the only available option. Biological aortic valve replacement is generally performed with prosthetic valves made from either bovine or porcine tissue. Porcine valves have been associated with a higher risk of reoperation, but no clinically significant difference in the risk of heart failure hospitalization or mortality has been found between the 2 valve types.^9^ Porcine valves may be more prone to degeneration, and it has been hypothesized that damaged valvular endothelium makes patients more vulnerable to endocarditis.^2^ Thus, porcine valves may be more susceptible to bacterial overgrowth. However, bovine and porcine prostheses tend to degenerate in different manners,^10^, ^11^ which may affect their vulnerability to microbial overgrowth. Whether porcine aortic valve prostheses carry a higher risk of endocarditis than bovine prostheses is unknown. Therefore, we conducted a national population‐based cohort study to evaluate the risk of endocarditis in patients who underwent SAVR with porcine and bovine bioprostheses.

## METHODS

### Study Design

This observational, nationwide, population‐based cohort study was performed according to the Reporting of Studies Conducted Using Observational Routinely Collected Health Data statement^12^ and the Strengthening the Reporting of Observational Studies in Epidemiology guidelines.^13^ The authors declare that all supporting data are available within the article (and its online supplementary files). The study was approved by the Swedish Ethical Review Authority (Registration number: 2020‐04967), and the requirement for informed consent was waived.

### Setting

All patients who underwent SAVR with a biological valve prosthesis in Sweden from January 1, 1997 to December 31, 2018 were included in the study. Aortic valve replacement is performed at 8 centers in Sweden. Follow‐up for endocarditis and survival ended on December 31, 2018 and March 17, 2020, respectively.

### Study Population

We included all patients who underwent SAVR in Sweden from 1997 to 2018. The exclusion criteria were multiple valve surgery, prior TAVR, prior cardiac surgery, undetermined prosthesis type, use of a stentless valve, and surgery in deep hypothermia.

### Exposure and Outcomes

The exposure was SAVR with implantation of a bovine bioprosthesis, and the control group comprised patients who underwent SAVR with implantation of a porcine bioprosthesis. The exposure was identified from the Swedish Web System for Enhancement and Development of Evidence‐Based Care in Heart Disease Evaluated According to Recommended Therapies (SWEDEHEART) register. The primary outcome measure was first‐time hospitalization for PVE obtained from the National Patient Register. The secondary outcomes were early (>90 days and <1 year after SAVR) and late (>1 year after SAVR) endocarditis as well as all‐cause mortality. A 90‐day washout period was added to the endocarditis definition to avoid misclassification of endocarditis indication for surgery as an endocarditis outcome. Data on early and late endocarditis were obtained from the National Patient Register, and data on mortality were obtained from the Swedish Cause of Death Register. The *International Classification of Diseases, Tenth Revision* (*ICD‐10*) codes to define endocarditis were I33.0, I33.9, I38.9, I39.8, and B37.6, and *International Classification of Diseases, Ninth Revision* (*ICD‐9*) codes were 421 and 391B.

### Data Sources

The study population and baseline characteristics were obtained from the SWEDEHEART register,^14^, ^15^, ^16^ which is a register with national coverage of all patients (including detailed operative and clinical information) who have undergone cardiac surgery in Sweden since 1992. The SWEDEHEART register was also used to identify patients with bovine or porcine bioprostheses. Further baseline data and information about endocarditis were obtained from the National Patient Register, which has complete national coverage of all hospital discharges since 1987 and is maintained by the Swedish National Board of Health and Welfare.^17^ The *ICD‐9* and *ICD‐10* codes were used to obtain baseline characteristics and information about infective endocarditis from the National Patient Register. The Longitudinal Integration Database for Health Insurance and Labor Market Study register,^18^ which is a national register maintained by Statistics Sweden, was used to collect data on socioeconomic variables. A unique personal identity number is assigned to all Swedish citizens at birth,^19^ and this number was used to cross‐link data from different Swedish registers at an individual level. The national registers used to form the database have been described in detail previously.^8^ Because of Swedish data protection laws, we are not able to share or to make the database available.

### Statistical Analysis

Baseline characteristics are described as mean and SD for continuous variables and as frequency and percentage for categorical variables. Time to event was defined as the time in days from the date of surgery to either the date of the event or the end of follow‐up on December 31, 2018. The Aalen‐Johansen estimator was used to estimate the crude cumulative incidence of endocarditis while accounting for the competing risk of death, and the Kaplan‐Meier estimator was used to estimate crude all‐cause mortality. Age‐ and sex‐adjusted incidence rates were estimated using a Poisson model. Regression standardization was used to adjust for differences in baseline characteristics between the groups.^20^ The standardized cumulative incidence of endocarditis was estimated using flexible hazard‐based regression standardization. We adjusted for the distribution of baseline characteristics and accounted for the competing risk of death, as previously described by Kipouro et al.^21^ Flexible parametric survival regression standardization was used to estimate standardized survival.^20^, ^22^ Statistical significance was decided at an α value of 5%. Model selection was performed using clinical subject‐matter knowledge and was guided by the Akaike information criterion. The final models used are presented in Data S1. We performed subgroup analyses by excluding patients who had a preoperative pacemaker or implantable cardioverter device, who had a history of preoperative drug abuse, or who underwent an operation for endocarditis. Statistical analyses and data management were performed using the R Programming language, version 4.3.0 (R Foundation for Statistical Computing, Vienna, Austria) and involved use of the survival, ggplot2, simputation, mexhaz, and rstpm2 packages. Code will be made available on reasonable request from the corresponding author.

### Missing Data

The following variables had missing data: left ventricular ejection fraction (15.6%), emergent operation (15.6%), body mass index (9.3%), estimated glomerular filtration rate (5.6%), educational level (1.4%), valve size (0.5%), and disposable family income (<0.1%). Missing data were handled using classification and regression tree imputation.^23^

## RESULTS

### Study Population

We included 21 022 patients who underwent SAVR with a bioprosthesis. Of these patients, 16 603 (79%) received a bovine bioprosthesis, and 4419 (21%) received a porcine bioprosthesis. The use of bovine prostheses increased during the study period. The number of bovine versus porcine prostheses used per year and the most commonly used valve prosthesis models are shown in Figure S1 and Table S1, respectively. The patients' mean age was 73 years, and 61% were men. Patients in the bovine group were younger (73 versus 75 years) and had a higher prevalence of men, hypertension, and diabetes as well as a higher educational level and household income. Patients in the porcine group had a higher prevalence of prior myocardial infarction, heart failure, chronic kidney disease, and concomitant coronary artery bypass grafting. The baseline characteristics in both groups are shown in Table 1.

**Table 1:** Baseline Characteristics of 21 022 Patients Who Underwent Bioprosthetic Aortic Valve Replacement in Sweden Between 1997 and 2018, Classified According to Xenograft Material

Variable	Overall	Bovine	Porcine
No.	21 022	16 603	4419
Age, mean (SD), y	73.0 (8.3)	72.5 (8.4)	75.2 (7.4)
Male sex	12 868 (61.2)	10 279 (61.9)	2589 (58.6)
Married	13 347 (63.5)	10 587 (63.8)	2760 (62.5)
Body mass index, kg/m^2^			
<18.5	204 (1.1)	166 (1.1)	38 (1.0)
18.5–24.9	6700 (35.1)	5181 (34.4)	1519 (38.0)
25–29.9	8101 (42.5)	6379 (42.3)	1722 (43.1)
>30	4068 (21.3)	3348 (22.2)	720 (18.0)
Education, y			
<10	9516 (45.9)	7287 (44.5)	2229 (51.3)
10–12	7510 (36.2)	6035 (36.8)	1475 (34.0)
>12	3710 (17.9)	3070 (18.7)	640 (14.7)
Household income			
Quartile 1 (lowest)	5248 (25.0)	3838 (23.1)	1410 (31.9)
Quartile 2	5260 (25.0)	3961 (23.9)	1299 (29.4)
Quartile 3	5246 (25.0)	4251 (25.6)	995 (22.5)
Quartile 4 (highest)	5262 (25.0)	4551 (27.4)	711 (16.1)
Non‐Nordic birth region	1182 (5.6)	941 (5.7)	241 (5.5)
LVEF, %			
<30	917 (5.2)	732 (5.1)	185 (5.4)
30–50	3886 (21.9)	3049 (21.3)	837 (24.4)
>50	12 933 (72.9)	10 521 (73.6)	2412 (70.2)
Prior myocardial infarction	3474 (16.5)	2628 (15.8)	846 (19.1)
Prior heart failure	4664 (22.2)	3573 (21.5)	1091 (24.7)
Prior atrial fibrillation	3754 (17.9)	2956 (17.8)	798 (18.1)
Pacemaker/ICD	534 (2.5)	445 (2.7)	89 (2.0)
Prior PCI	1768 (8.4)	1505 (9.1)	263 (6.0)
Hyperlipidemia	4239 (20.2)	3510 (21.1)	729 (16.5)
Hypertension	10 628 (50.6)	8758 (52.7)	1870 (42.3)
Peripheral vascular disease	2515 (12.0)	2008 (12.1)	507 (11.5)
eGFR, mL/min per 1.73 m^2^			
<30	567 (2.9)	427 (2.7)	140 (3.3)
30–44	1706 (8.6)	1303 (8.3)	403 (9.5)
45–59	4058 (20.4)	3085 (19.8)	973 (23.0)
>60	13 516 (68.1)	10 805 (69.2)	2711 (64.1)
COPD	2156 (10.3)	1702 (10.3)	454 (10.3)
Diabetes	4395 (20.9)	3565 (21.5)	830 (18.8)
Prior stroke	2435 (11.6)	1915 (11.5)	520 (11.8)
History of cancer	3311 (15.8)	2656 (16.0)	655 (14.8)
Hepatic disease	311 (1.5)	253 (1.5)	58 (1.3)
Alcohol dependence	559 (2.7)	463 (2.8)	96 (2.2)
Drug abuse	120 (0.6)	97 (0.6)	23 (0.5)
Opioid abuse	28 (0.1)	25 (0.2)	3 (0.1)
Prior endocarditis	322 (1.5)	268 (1.6)	54 (1.2)
Surgery for endocarditis	705 (3.4)	602 (3.6)	103 (2.3)
Period of surgery, y			
1997–2002	3335 (15.9)	2181 (13.1)	1154 (26.1)
2003–2008	5390 (25.6)	3618 (21.8)	1772 (40.1)
2009–2013	6168 (29.3)	4837 (29.1)	1331 (30.1)
2014–2018	6129 (29.2)	5967 (35.9)	162 (3.7)
Size, mm			
18–21	7073 (33.8)	5853 (35.4)	1220 (27.8)
22–23	8126 (38.9)	6285 (38.0)	1841 (41.9)
≥25	5717 (27.3)	4384 (26.5)	1333 (30.3)
Concomitant CABG	8486 (40.4)	6292 (37.9)	2194 (49.6)
Ascending aortic surgery	1633 (7.8)	1407 (8.5)	226 (5.1)
Emergent operation	294 (1.7)	242 (1.7)	52 (1.5)
Model group			
Perimount	13 276 (63.2)	13 276 (80.0)	0 (0.0)
Hancock/Mosaic	1628 (7.7)	0 (0.0)	1628 (36.8)
Biocor/Epic	2612 (12.4)	0 (0.0)	2612 (59.1)
Mitroflow/Crown	1977 (9.4)	1977 (11.9)	0 (0.0)
Soprano	982 (4.7)	982 (5.9)	0 (0.0)
Other	547 (2.6)	368 (2.2)	179 (4.1)

Data are given as number (percentage) unless otherwise noted. CABG indicates coronary artery bypass grafting; COPD, chronic obstructive pulmonary disease; eGFR, estimated glomerular filtration rate; ICD, implantable cardioverter‐defibrillator; LVEF, left ventricular ejection fraction; and PCI, percutaneous coronary intervention.

### Prosthetic Valve Endocarditis

The mean follow‐up time was 6.0 years (maximum, 22 years). In total, 910 patients were hospitalized for infective endocarditis: 690 (4.2%) in the bovine group and 220 (5.0%) in the porcine group. The crude incidence rate of PVE was 0.72% (95% CI, 0.68%–0.77%) per person‐year overall, and 0.75% (95% CI, 0.69%–0.81%) and 0.65% (95% CI, 0.57%–0.74%) per person‐year in the bovine and porcine groups, respectively. The crude cumulative incidence for PVE at 5, 10, and 15 years was 3.3% versus 2.7%, 5.5% versus 4.7%, and 6.4% versus 5.7% in the bovine versus porcine group, respectively.

**Figure 1:**
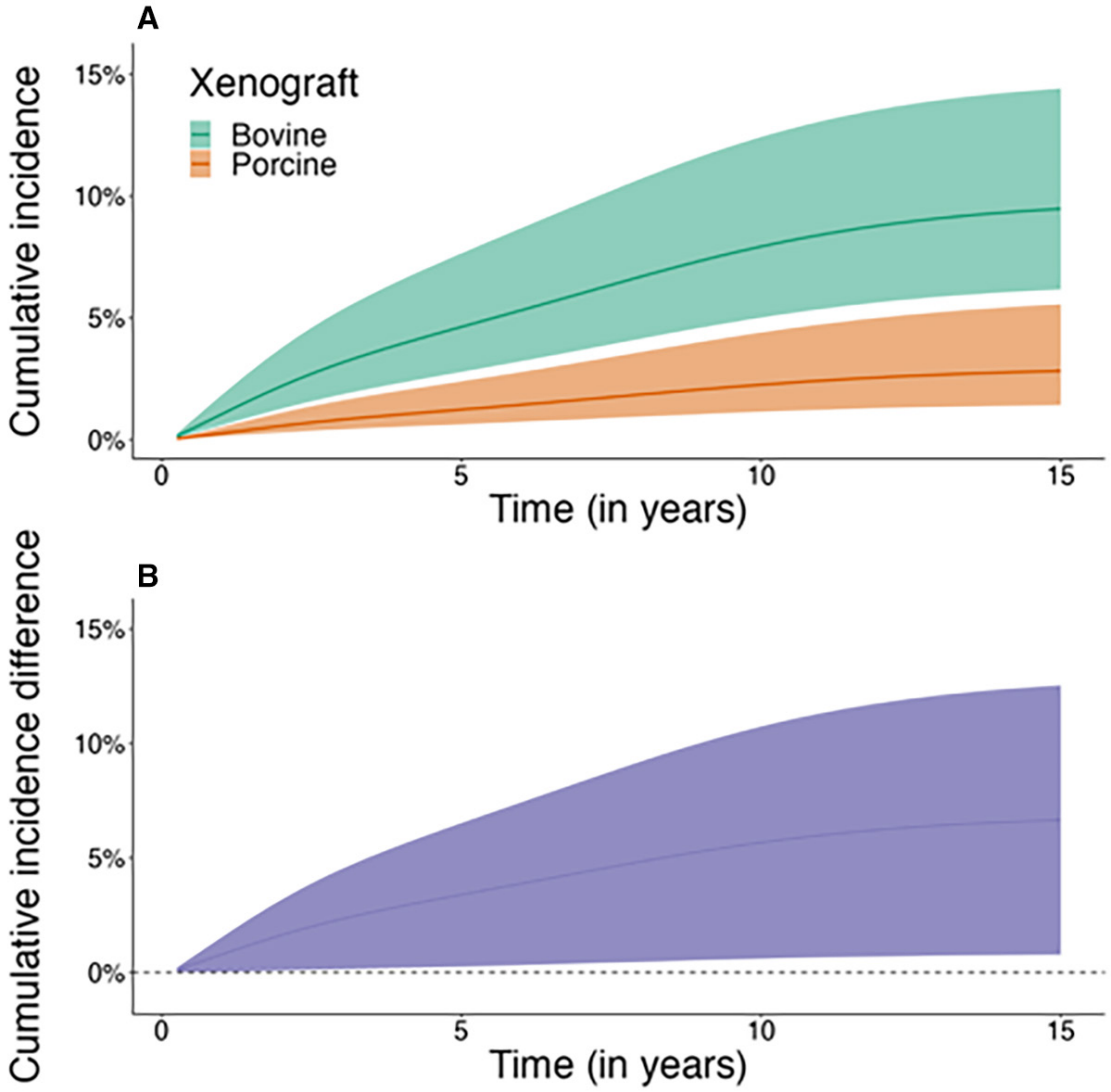
Regression standardized cumulative incidence of prosthetic valve endocarditis. Regression standardized cumulative incidence and 95% CI of prosthetic valve endocarditis in patients who underwent surgical aortic valve replacement with bovine vs porcine bioprostheses (**A**) and estimated difference and 95% CI in prosthetic valve endocarditis between patients with bovine vs porcine bioprostheses (**B**).

After regression standardization, the risk of PVE was higher in the bovine than in the porcine group. The estimated cumulative incidence of PVE at 15 years was 9.5% (95% CI, 6.2%–14.4%) versus 2.8% (95% CI, 1.4%–5.6%) in the bovine and porcine group, respectively. The absolute risk difference between the groups at 15 years was 6.7% (95% CI, 0.8%–12.5%). The crude and age‐ and sex‐adjusted incidence rates of PVE are shown in Table S2; the crude cumulative incidence at 5, 10, and 15 years is shown in Table 2; and the adjusted cumulative incidence and absolute differences between the groups at 5, 10, and 15 years are shown in Figure 1 and Table 3, respectively.

**Table 2:** Crude Cumulative Incidence for Endocarditis and All‐Cause Mortality According to Xenograft Material

Variable	Events/PY	1 y	5 y	10 y	15 y
		Cumulative incidence, % (95% CI)			
Endocarditis					
Bovine	690/92 069	0.9 (0.8–1.0)	3.3 (3.0–3.6)	5.5 (5.0–5.9)	6.4 (5.8–6.9)
Porcine	220/33 930	0.7 (0.4–0.9)	2.7 (2.2–3.2)	4.7 (4.0–5.3)	5.7 (5.0–6.5)
All‐cause mortality					
Bovine	7180/105 843	6 (6–6)	20 (20–21)	50 (49–51)	79 (78–80)
Porcine	3053/36 474	7 (6–7)	23 (22–24)	54 (52–56)	80 (78–81)
Late endocarditis					
Bovine	547/92 069	0.0 (0.0–0.0)	2.4 (2.1–2.7)	4.6 (4.2–5.0)	5.5 (5.0–5.9)
Porcine	191/33 930	0.0 (0.0–0.0)	2.1 (1.6–2.5)	4.0 (3.4–4.6)	5.1 (4.3–5.8)
Early endocarditis					
Bovine	143/15 209	0.9 (0.8–1.0)	NA	NA	NA
Porcine	29/4191	0.7 (0.4–0.9)	NA	NA	NA

Endocarditis using Aalen‐Johansen estimator accounting for the competing risk of death. NA indicates not applicable; and PY, person‐years.

**Table 3:** Regression Standardized Cumulative Incidence and Differences for Endocarditis and All‐Cause Mortality at 5, 10, and 15 Years for Patients Who Underwent Bioprosthetic Aortic Valve Replacement in Sweden Between 1997 and 2018

Variable	1 y	5 y	10 y	15 y
Endocarditis				
Bovine	1.1 (0.6 to 1.8)	4.6 (2.8 to 7.7)	7.9 (5 to 12.4)	9.5 (6.2 to 14.4)
Porcine	0.3 (0.1 to 0.5)	1.2 (0.6 to 2.4)	2.3 (1.2 to 4.4)	2.8 (1.4 to 5.6)
Difference bovine vs porcine	0.8 (0.0 to 1.5)	3.4 (0.3 to 6.5)	5.7 (0.6 to 10.7)	6.7 (0.8 to 12.5)
All‐cause mortality				
Bovine	6 (6 to 7)	23 (21 to 24)	47 (45 to 49)	74 (73 to 76)
Porcine	5 (4 to 6)	19 (15 to 22)	40 (34 to 46)	67 (61 to 74)
Difference bovine vs porcine	1.4 (−0.1 to 2.9)	4.4 (−0.3 to 9.0)	7.0 (−0.5 to 14.6)	6.9 (−0.8 to 14.5)
Late endocarditis				
Bovine	0.2 (0.1 to 0.4)	4.2 (2.2 to 8.1)	8.1 (4.6 to 14.2)	9.9 (5.8 to 16.5)
Porcine	0 (0 to 0.1)	0.8 (0.4 to 1.6)	1.7 (0.9 to 3.3)	2.2 (1.1 to 4.3)
Difference bovine vs porcine	0.1 (0.0 to 0.3)	3.4 (0.1 to 6.7)	6.4 (0.7 to 12.1)	7.7 (1.1 to 14.2)

Adjusted by regression standardization. A detailed description and documentation for included covariates are available in Data S1.

### Early and Late PVE

Early endocarditis occurred in 172 (19%) of the 910 patients with endocarditis: 143 (83%) in the bovine group and 29 (17%) in the porcine group. Late endocarditis occurred in 738 (81%) of the 910 patients with endocarditis: 547 (74%) in the bovine group and 191 (26%) in the porcine group. The crude incidence rate of early and late PVE was 0.94% (95% CI, 0.79%–1.11%) and 0.59% (95% CI, 0.55%–0.65%) per person‐year in the bovine group and 0.69% (95% CI, 0.46%–0.99%) and 0.56% (95% CI, 0.49%–0.65%) in the porcine group.

After regression standardization, the risk of early endocarditis was higher in patients with bovine prostheses (absolute risk difference, 0.8% [95% CI, 0.0%–1.5%]). At 15 years after surgery, the estimated cumulative incidence of late PVE was 9.9% (95% CI, 5.8%–16.5%) versus 2.2% (95% CI, 1.1%–4.3%) in the bovine and porcine group, respectively. This difference was statistically significant, with an absolute risk difference at 15 years of 7.7% (95% CI, 1.1%–14.2%). The crude and age‐ and sex‐adjusted incidence rates of early and late PVE are shown in Table S2; the crude cumulative incidence rates at 5, 10, and 15 years are shown in Table 2; and the adjusted cumulative incidence and absolute differences between the groups at 5, 10, and 15 years are shown in Figure S2 and Table 3, respectively.

### All‐Cause Mortality

In total, 10 233 (49%) patients died during follow‐up: 7180 (43%) in the bovine group and 3053 (69%) in the porcine group. The 30‐day mortality rate after SAVR was 3.0% in both groups, and the 30‐day mortality rate after the initial diagnosis of PVE was 17% in patients with bovine prostheses and 21% in patients with porcine prostheses. The crude incidence rate of death was 6.8% (95% CI, 6.6%–6.9%) and 8.4% (95% CI, 8.1%–8.7%) per person‐year in the bovine and porcine groups, respectively. The crude cumulative incidence of death at 5, 10, and 15 years is 20% versus 23%, 50% versus 54%, and 79% versus 80% in the bovine versus porcine group, respectively.

**Figure 2:**
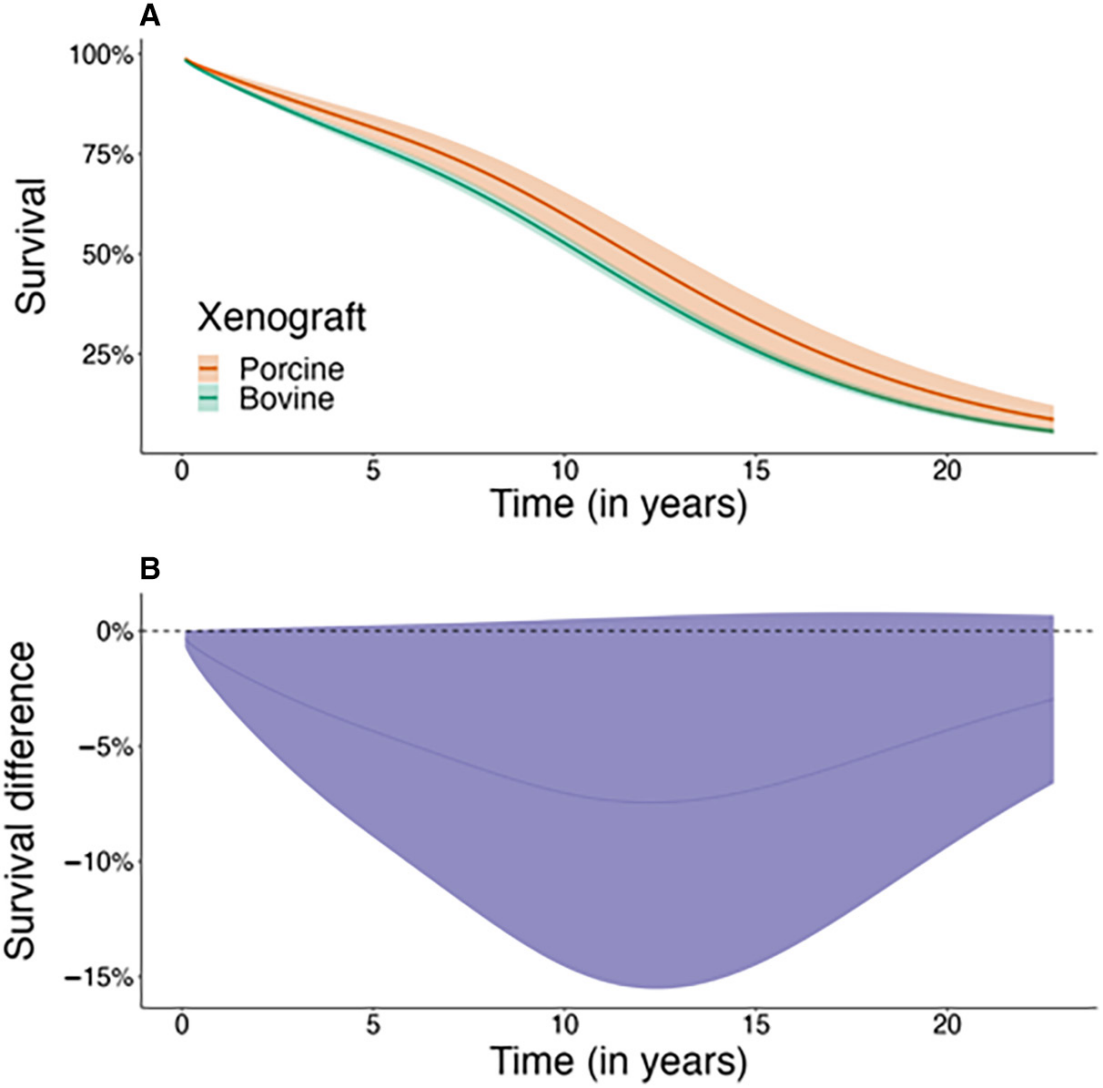
Regression standardized survival. Regression standardized survival and 95% CI of survival in patients who underwent surgical aortic valve replacement with bovine vs porcine bioprostheses (**A**) and estimated difference and 95% CI in survival between patients with bovine vs porcine bioprostheses (**B**).

After regression standardization, there was no statistically significant difference in the risk of death between the groups. The estimated cumulative incidence of death at 15 years was 74% (95% CI, 73%–76%) and 67% (95% CI, 61%–74%) in the bovine and porcine groups, respectively. The absolute risk difference between the groups at 15 years was 6.9% (95% CI, −0.8% to 14.5%). The crude and age‐ and sex‐adjusted incidence rates of death are shown in Table S2; the crude cumulative incidence at 5, 10, and 15 years is shown in Table 2; and the adjusted cumulative incidence and absolute differences between the groups at 5, 10, and 15 years are shown in Figure 2 and Table 3, respectively.

### Subgroup Analyses

Separate analyses excluding patients with intravenous drug use, patients with cardiac implantable electronic devices, and patients who had undergone surgery for endocarditis showed results similar to those of the main analyses. The Aalen‐Johansen and Kaplan‐Meier curves and associated number at risk tables for prosthetic valve endocarditis and survival are presented in Figures S3 and S4, respectively.

## DISCUSSION

In this nationwide, population‐based cohort study, we found a higher risk of endocarditis in patients who underwent SAVR with a bovine compared with a porcine bioprosthesis, with an absolute risk difference of 7% at 15 years. There was no difference in the risk of all‐cause mortality between the groups. The incidence of PVE was highest during the first year after SAVR and remained stable thereafter.

In a previous study from our group, we found an annual 0.57% incidence of endocarditis and a higher risk of PVE in patients with biological than mechanical valve prostheses.^6^ The finding that PVE is more common in patients with biological valves is supported by other studies.^7^ To our knowledge, no prior studies have directly compared the risk of endocarditis in patients who underwent SAVR with bovine versus porcine bioprostheses. However, in the TAVR population, the risk of endocarditis between patients with self‐expandable versus balloon‐expandable prostheses has been investigated.^24^

Regueiro et al^24^ found no difference in the rate of endocarditis among 6255 patients who underwent TAVR with a self‐expandable (n=2719; all porcine prostheses) versus balloon‐expandable prosthesis (n=3536; all bovine prostheses). The cumulative incidence of PVE was 0.95% and 1.25% at 1 year after TAVR in the self‐expandable (porcine) and balloon‐expandable (bovine) groups, respectively (*P*=0.33). In the present study, we also found a higher risk of endocarditis in patients with bovine compared with porcine bioprostheses. Notably, the 1‐year cumulative incidence of endocarditis was slightly lower in our cohort who underwent SAVR (0.7% for porcine prostheses and 0.9% for bovine prostheses). However, the prostheses used in SAVR and TAVR are anatomically different, as are the study populations who undergo TAVR and SAVR. Although TAVR prostheses often are made from pericardial tissue irrespective of xenograft origin, SAVR valves are often made from bovine pericardial tissue or porcine valve tissue.^25^ This means that differences between SAVR valves may be attributable to anatomic rather than xenograft origin. Therefore, these differences should be interpreted with caution.

Hickey et al^26^ analyzed 38 040 patients who underwent SAVR in England and Wales with bovine or porcine bioprostheses from 2003 to 2013 and found no difference in survival or reintervention‐free survival. They reported a 10‐year survival rate of 49% in the bovine group and 50% in the porcine group. These results are consistent with our findings, with a corresponding 10‐year survival rate of 50% and 46% for patients with bovine and porcine prostheses, respectively. However, our study adds important information about the risk of PVE after SAVR with bovine and porcine bioprostheses. Furthermore, and in agreement with prior studies,^27^ we found a high mortality rate after PVE (18% within 30 days after the initial diagnosis of PVE). Andreas et al^28^ investigated long‐term survival and valve‐related adverse events in 458 patients who underwent SAVR with either a Medtronic Mosaic (porcine) or a Carpentier Edwards Perimount Magna (bovine) prosthesis. In line with our results, they found similar long‐term survival between the groups. They also reported similar rates of endocarditis, but only 2 (0.3%) patients in the porcine group and 9 (0.7%) patients in the bovine group had endocarditis during follow‐up.^28^ In our study, 690 (4.1%) patients in the bovine group and 220 (5.0%) patients in the porcine group had endocarditis. The discrepancy in the cumulative incidence of PVE between the studies can probably be explained by the larger number of patients and the longer follow‐up in our study. Johnston et al^10^ followed up 12 569 patients who underwent SAVR with a Carpentier Edwards Perimount aortic valve prosthesis, which is made from bovine pericardium, and found a 20‐year probability of reoperation for endocarditis of 1.4%. In our study, the 15‐year crude risk of endocarditis was 6.4% in the bovine group. However, Johnston et al^10^ counted only valve explantation attributable to endocarditis, whereas we counted all cases of hospitalization for endocarditis, regardless of treatment strategy.

Prior studies have shown a higher risk of reoperation in patients with porcine compared with bovine bioprostheses.^9^, ^29^ This could be related to an increased risk of structural valve deterioration in these patients, which could possibly predispose porcine valves to bacterial overgrowth. However, different mechanisms have been suggested for valve failure in porcine and bovine prostheses; porcine prostheses tend to fail because of cusp tears and valve insufficiency, whereas bovine prostheses undergo gradual degenerative changes that cause increasing stenosis.^10^, ^11^ Gradual degeneration rather than cusp tears or valve incompetence might make bovine prostheses more prone to bacterial overgrowth. This may explain the higher risk of endocarditis associated with bovine prostheses found in this study.

The incidence rate of PVE after SAVR is ≈0.5% to 1.0% per year,^6^, ^30^ and similar rates were reported after TAVR^31^, ^32^, ^33^ and mitral valve replacement.^34^ Our results are consistent with the incidence found in prior studies (0.7% per year). In the current era in which TAVR is performed in younger patients with lower surgical risk, the incidence of PVE in patients with biological valve prostheses is of utmost interest, and it is important to investigate differences in valve‐related complications between different types of valve prostheses. Although the results of our study cannot be directly extrapolated to patients who undergo TAVR, our study adds important information about valve‐related complications in a nationwide, large patient cohort with long follow‐up.

### Strengths and Limitations

This study has several strengths. First, the nationwide and population‐based design increases both internal and external validity. Second, internal validity is high because of the high quality of the Swedish registers. Third, follow‐up was complete, and the study included many patients. However, this study also has some limitations. Because of the observational study design, there may have been unmeasured or unknown factors that could not be adjusted for, thereby influencing the results (ie, residual confounding). For example, we did not have information about frailty, dental hygiene, preoperative immune deficiency, corticosteroid treatment, severity of diabetes, the invasiveness of the infection, the microbial organisms involved in PVE, or the antibiotic regimen. However, we did have information about many clinically important comorbidities and social factors. To avoid misclassification of endocarditis indication for surgery as an endocarditis outcome, we added a 90‐day washout period to the endocarditis definition, which may have led to an underestimation of the PVE incidence. Furthermore, we might have underestimated or overestimated the incidence of PVE because we had to rely on the information in the *ICD‐10* codes, and some patients may not have been correctly diagnosed. However, this should have affected both groups equally (nondifferential misclassification) and therefore was unlikely to have affected the results. It was not possible to distinguish whether PVE affected the aortic valve prosthesis, another valve, or a cardiac implantable electronic device. However, a subgroup analysis was performed after excluding all patients with a cardiac implantable electronic device, and it showed results similar to those of the main analyses.

## CONCLUSIONS

In this nationwide, population‐based cohort study, we found a higher risk of endocarditis in patients who received a bovine compared with a porcine valve prosthesis after SAVR. There was no difference in the risk of all‐cause mortality between the groups. The incidence of PVE was highest the first year after SAVR and was thereafter stable during a follow‐up of up to 22 years. Our study adds novel and clinically relevant information about the long‐term prognosis in patients who undergo aortic valve replacement with bovine or porcine bioprostheses.

## Sources of Funding

This work received support from the Swedish Heart‐Lung Foundation (grants 20220364 and 20190570 to Dr Glaser and grant 20190533 to Dr Sartipy), Region Stockholm (ALF Project) (grants FoUI‐954 783 and FoUI‐961 871 to Dr Glaser and grant FoUI‐962 048 to Dr Sartipy), Region Stockholm clinical postdoctoral appointment (grant FoUI‐955 489 to Dr Glaser), the Swedish Society of Medicine (grant SLS‐934749 to Dr Glaser), the Eva and Oscar Ahrén Research Foundation (to Dr Glaser), the Seraphim Hospital Foundation (to Dr Glaser), the Magnus Bergvall Foundation (grants 2022‐177 and 2021‐04333 to Dr Glaser), the Mats Kleberg Foundation (grant 2022‐119 to Dr Glaser), Karolinska Institutet Foundations and Funds (grant 2022‐01575 to Dr Glaser), and a donation from Mr Fredrik Lundberg and the Schörling Foundation. The supporting bodies had no role in the design and conduct of the study; collection, management, analysis, and interpretation of the data; preparation, review, or approval of the manuscript; and decision to submit the manuscript for publication.

## Disclosures

None.

## Supporting information

Data S1
Tables S1–S2
Figures S1–S4
References^35^, ^36^, ^37^, ^38^

## References

[1] Selton‐Suty C , Celard M , Le Moing V , Doco‐Lecompte T , Chirouze C , Iung B , Strady C , Revest M , Vandenesch F , Bouvet A , et al. Preeminence of *Staphylococcus aureus* in infective endocarditis: a 1‐year population‐based survey. Clin Infect Dis. 2012;54:1230–1239. doi: 10.1093/cid/cis199 22492317

[2] Iung B , Duval X . Infective endocarditis: innovations in the management of an old disease. Nat Rev Cardiol. 2019;16:623–635. doi: 10.1038/s41569-019-0215-0 31175343

[3] Habib G , Lancellotti P , Antunes MJ , Bongiorni MG , Casalta JP , Del Zotti F , Dulgheru R , El Khoury G , Erba PA , Iung B , et al. 2015 ESC Guidelines for the management of infective endocarditis: the Task Force for the Management of Infective Endocarditis of the European Society of Cardiology (ESC). Endorsed by: European Association for Cardio‐Thoracic Surgery (EACTS), the European Association of Nuclear Medicine (EANM). Eur Heart J. 2015;36:3075–3128. doi: 10.1093/eurheartj/ehv319 26320109

[4] Murdoch DR , Corey GR , Hoen B , Miro JM , Fowler VG Jr , Bayer AS , Karchmer AW , Olaison L , Pappas PA , Moreillon P , et al. Clinical presentation, etiology, and outcome of infective endocarditis in the 21st century: the International Collaboration on Endocarditis–Prospective Cohort Study. Arch Intern Med. 2009;169:463–473. doi: 10.1001/archinternmed.2008.603 19273776 PMC3625651

[5] Ternhag A , Cederstrom A , Törner A , Westling K . A nationwide cohort study of mortality risk and long‐term prognosis in infective endocarditis in Sweden. PLoS One. 2013;8:e67519. doi: 10.1371/journal.pone.0067519 23861768 PMC3704638

[6] Glaser N , Jackson V , Holzmann MJ , Franco‐Cereceda A , Sartipy U . Prosthetic valve endocarditis after surgical aortic valve replacement. Circulation. 2017;136:329–331. doi: 10.1161/CIRCULATIONAHA.117.028783 28716834

[7] Brennan JM , Edwards FH , Zhao Y , O'Brien S , Booth M , Dokholyan R , Douglas P , Peterson E . Long‐term safety and effectiveness of mechanical versus biologic aortic valve prostheses in older patients: results from the Society of Thoracic Surgeons Adult Cardiac Surgery National Database. Circulation. 2013;127:1647–1655. doi: 10.1161/CIRCULATIONAHA.113.002003 23538379

[8] Glaser N , Jackson V , Holzmann MJ , Franco‐Cereceda A , Sartipy U . Aortic valve replacement with mechanical vs. biological prostheses in patients aged 50–69 years. Eur Heart J. 2016;37:2658–2667. doi: 10.1093/eurheartj/ehv580 26559386

[9] Persson M , Glaser N , Franco‐Cereceda A , Nilsson J , Holzmann MJ , Sartipy U . Porcine vs bovine bioprosthetic aortic valves: long‐term clinical results. Ann Thorac Surg. 2021;111:529–535. doi: 10.1016/j.athoracsur.2020.05.126 32693042

[10] Johnston DR , Soltesz EG , Vakil N , Rajeswaran J , Roselli EE , Sabik JF , Smedira NG , Svensson LG , Lytle BW , Blackstone EH . Long‐term durability of bioprosthetic aortic valves: implications from 12,569 implants. Ann Thorac Surg. 2015;99:1239–1247. doi: 10.1016/j.athoracsur.2014.10.070 25662439 PMC5132179

[11] Arsalan M , Walther T . Durability of prostheses for transcatheter aortic valve implantation. Nat Rev Cardiol. 2016;13:360–367. doi: 10.1038/nrcardio.2016.43 27053461

[12] Benchimol EI , Smeeth L , Guttmann A , Harron K , Moher D , Petersen I , Sorensen HT , von Elm E , Langan SM . The REporting of studies Conducted using Observational Routinely‐collected health Data (RECORD) statement. PLoS Med. 2015;12:e1001885. doi: 10.1371/journal.pmed.1001885 26440803 PMC4595218

[13] von Elm E , Altman DG , Egger M , Pocock SJ , Gotzsche PC , Vandenbroucke JP . The Strengthening the Reporting of Observational Studies in Epidemiology (STROBE) statement: guidelines for reporting observational studies. J Clin Epidemiol. 2008;61:344–349. doi: 10.1016/j.jclinepi.2007.11.008 18313558

[14] Emilsson L , Lindahl B , Koster M , Lambe M , Ludvigsson JF . Review of 103 Swedish healthcare quality registries. J Intern Med. 2015;277:94–136. doi: 10.1111/joim.12303 25174800

[15] Jernberg T , Attebring MF , Hambraeus K , Ivert T , James S , Jeppsson A , Lagerqvist B , Lindahl B , Stenestrand U , Wallentin L . The Swedish Web‐system for Enhancement and Development of Evidence‐based care in Heart disease Evaluated According to Recommended Therapies (SWEDEHEART). Heart. 2010;96:1617–1621. doi: 10.1136/hrt.2010.198804 20801780

[16] Vikholm P , Ivert T , Nilsson J , Holmgren A , Freter W , Ternstrom L , Ghaidan H , Sartipy U , Olsson C , Granfeldt H , et al. Validity of the Swedish cardiac surgery registry. Interact Cardiovasc Thorac Surg. 2018;27:67–74. doi: 10.1093/icvts/ivy030 29452368

[17] Ludvigsson JF , Andersson E , Ekbom A , Feychting M , Kim J , Reuterwall C , Heurgren M , Olausson Otterblad P . External review and validation of the Swedish national inpatient register. BMC Public Health. 2011;11:450. doi: 10.1186/1471-2458-11-450 21658213 PMC3142234

[18] Ludvigsson JF , Svedberg P , Olen O , Bruze G , Neovius M . The longitudinal integrated database for health insurance and labour market studies (LISA) and its use in medical research. Eur J Epidemiol. 2019;34:423–437. doi: 10.1007/s10654-019-00511-8 30929112 PMC6451717

[19] Ludvigsson JF , Otterblad‐Olausson P , Pettersson BU , Ekbom A . The Swedish personal identity number: possibilities and pitfalls in healthcare and medical research. Eur J Epidemiol. 2009;24:659–667. doi: 10.1007/s10654-009-9350-y 19504049 PMC2773709

[20] Syriopoulou E , Wasterlid T , Lambert PC , Andersson TM . Standardised survival probabilities: a useful and informative tool for reporting regression models for survival data. Br J Cancer. 2022;127:1808–1815. doi: 10.1038/s41416-022-01949-6 36050446 PMC9643385

[21] Kipourou DK , Charvat H , Rachet B , Belot A . Estimation of the adjusted cause‐specific cumulative probability using flexible regression models for the cause‐specific hazards. Stat Med. 2019;38:3896–3910. doi: 10.1002/sim.8209 31209905 PMC6771712

[22] Liu XR , Pawitan Y , Clements M . Parametric and penalized generalized survival models. Stat Methods Med Res. 2018;27:1531–1546. doi: 10.1177/0962280216664760 27587596

[23] Breiman L . Classification and Regression Trees. Chapman & Hall; 2017. doi: 10.1201/9781315139470

[24] Regueiro A , Linke A , Latib A , Ihlemann N , Urena M , Walther T , Husser O , Herrmann H , Nombela‐Franco L , Asim C , et al. Infective endocarditis following transcatheter aortic valve replacement: comparison of balloon‐ versus self‐expandable valves. Circ Cardiovasc Interv. 2019;12:e007938. doi: 10.1161/CIRCINTERVENTIONS.119.007938 31694412

[25] Persson M , Glaser N , Nilsson J , Friberg O , Franco‐Cereceda A , Sartipy U . Comparison of long‐term performance of bioprosthetic aortic valves in Sweden from 2003 to 2018. JAMA Netw Open. 2022;5:e220962. doi: 10.1001/jamanetworkopen.2022.0962 35254431 PMC8902647

[26] Hickey GL , Grant SW , Bridgewater B , Kendall S , Bryan AJ , Kuo J , Dunning J . A comparison of outcomes between bovine pericardial and porcine valves in 38,040 patients in England and Wales over 10 years. Eur J Cardiothorac Surg. 2015;47:1067–1074. doi: 10.1093/ejcts/ezu307 25189704

[27] Edlin P , Westling K , Sartipy U . Long‐term survival after operations for native and prosthetic valve endocarditis. Ann Thorac Surg. 2013;95:1551–1556. doi: 10.1016/j.athoracsur.2013.03.006 23562467

[28] Andreas M , Wallner S , Ruetzler K , Wiedemann D , Ehrlich M , Heinze G , Binder T , Moritz A , Hiesmayr MJ , Kocher A , et al. Comparable long‐term results for porcine and pericardial prostheses after isolated aortic valve replacement. Eur J Cardiothorac Surg. 2015;48:557–561. doi: 10.1093/ejcts/ezu466 25527170 PMC4573977

[29] Webb J , Parkin D , Tondel K , Simitsis P , Roxburgh J , Chambers JB . A comparison of early redo surgery rates in Mosaic porcine and Perimount bovine pericardial valves. Eur J Cardiothorac Surg. 2018;54:724–728. doi: 10.1093/ejcts/ezy113 29579171

[30] Varstela E . Personal follow‐up of 100 aortic valve replacement patients for 1081 patient years. Ann Chir Gynaecol. 1998;87:205–212. 9825065

[31] Bjursten H , Rasmussen M , Nozohoor S , Gotberg M , Olaison L , Ruck A , Ragnarsson S . Infective endocarditis after transcatheter aortic valve implantation: a nationwide study. Eur Heart J. 2019;40:3263–3269. doi: 10.1093/eurheartj/ehz588 31433472 PMC6911164

[32] Regueiro A , Linke A , Latib A , Ihlemann N , Urena M , Walther T , Husser O , Herrmann HC , Nombela‐Franco L , Cheema AN , et al. Association between transcatheter aortic valve replacement and subsequent infective endocarditis and in‐hospital death. JAMA. 2016;316:1083–1092. doi: 10.1001/jama.2016.12347 27623462

[33] Stortecky S , Heg D , Tueller D , Pilgrim T , Muller O , Noble S , Jeger R , Toggweiler S , Ferrari E , Taramasso M , et al. Infective endocarditis after transcatheter aortic valve replacement. J Am Coll Cardiol. 2020;75:3020–3030. doi: 10.1016/j.jacc.2020.04.044 32553254

[34] Lazam S , Vanoverschelde JL , Tribouilloy C , Grigioni F , Suri RM , Avierinos J‐F , de Meester C , Barbieri A , Rusinaru D , Russo A , et al. Twenty‐year outcome after mitral repair versus replacement for severe degenerative mitral regurgitation: analysis of a large, prospective, multicenter, international registry. Circulation. 2017;135:410–422. doi: 10.1161/CIRCULATIONAHA.116.023340 27899396

[35] Rothman KJ , Lash TL , VanderWeele TJ , Haneuse S . Modern Epidemiology. 4th ed. Wolters Kluwer; 2021. 10.1007/s10654-021-00778-w PMC8416883 34216355

[36] Hernán MA , Robins JM . Causal Inference: What If. Chapman & Hall/CRC; 2020. Accessed July 31, 2023. https://www.hsph.harvard.edu/miguel‐hernan/causal‐inference‐book/

[37] Sjölander A . Regression standardization with the R package stdReg. Eur J Epidemiol. 2016;31:563–574. doi: 10.1007/s10654-016-0157-3 27179798

[38] van Buuren S . Flexible Imputation of Missing Data. 2nd ed. Chapman & Hall/CRC; 2021.

