# Supplementary material for: Prosthetic Valve Endocarditis After Aortic Valve Replacement With Bovine Versus Porcine Bioprostheses
Source: J Am Heart Assoc. 2023 Dec 29;13(1):e031387. doi: 10.1161/JAHA.123.031387 (PMC10863842; doi:10.1161/JAHA.123.031387)
Supplement: Supplementary file 1 — Data S1 Tables S1–S2 Figures S1–S4 References 35 , 36 , 37 , 38 [file JAH3-13-e031387-s001.pdf]

SUPPLEMENTAL MATERIAL

Data S1. Supplemental Methods.

Regression standardization

A common way in observational studies to obtain confounder adjusted results in survival analysis is to use propensity score matching or inverse probability of treatment weighting methods. These methods involve constructing a model where the exposure (or treatment) is the response variable, and potential confounders are included as covariates. By matching or weighting on the (inverse) risk of belonging to one exposure group or the other, this can achieve balance of the distribution of the covariates between the constructed groups.

An alternative to this is regression standardization, also known as g-estimation using the parametric g-formula. This method instead models the outcome as the dependent variable and include the exposure and potential confounders as covariates. This model can then be used to predict the outcome for all individuals in the population. This step is done two times for a binary exposure, first setting all individuals exposure to the first level, and then setting all individuals exposure to the second level. Then, the mean of the outcome across the population is calculated. This can be interpreted as the expected outcome if the entire population would have treatment level X, versus the expected outcome if the entire population had treatment level Y, while all other covariates are held constant. While it is trivial to calculate the population average (although cumbersome in a survival setting with many different time points), the standard error calculations are bit more complex and benefit from computational support. This method has been implemented in the standsurv and stpm2 command in Stata, and in the rstpm2 and marginaleffects package in R, among others.

The interpretation of regression standardization is attractive when investigating the effect of xenograft material while accounting for other valve model effects. It would be hard to include valve model as a covariate when trying to create a model for the exposure, in this case xenograft material, as the valve model would be sufficient to predict the exposure rendering balance on the other covariates impossible. However, by modelling on the outcome and then including xenograft as exposure, and valve model as a covariate, regression standardization estimates what would happen if the entire population would have had a bovine valve (albeit the same valve model, with all other characteristics intact), versus if the entire population would have had a porcine valve (with the same valve model with all other characteristics intact).

For further reading, we recommend Rothman, Hernán and Robins, and Sjölander.[35-37]

Classification and regression tree imputation

The Classification and regression tree (CART) algorithm was first developed in the 1980s by Breiman and colleagues and has seen a revival as a popular machine learning algorithm. It is used to predict, estimate, or classify variables based on other variables, a characteristic that can be used to impute estimated values in case of missing data.

The algorithm works by classifying discrete variables using a classical classification tree based on Gini impurity and estimating continuous variables using a regression model and perform splits in the tree based on minimizing the residual sum of squares.

It is an attractive method as is can handle binary, categorical, and continuous variables both as predictors and as classification targets and is robust against outliers. The method has been implemented in R in the rpart, simputation, and mice packages.

For further reading, we recommend Buuren.[38]

Model selection

Model selection for the regression standardization models were performed using clinical subject matter knowledge and was informed using the Akaike information criterion. Continuous variables were tested using polynomials and splines during the selection process.

Endocarditis

The final model for endocarditis used a 3rd degree exponential B-spline with 2 interior knots at the 1/3 and 2/3 quantiles of the follow-up time for the baseline hazard. Age was included as a natural spline with four degrees of freedom. Year of surgery, body mass index and estimated glomerular filtration rate were included as natural splines with three degrees of freedom. The other included covariates were patient sex, operating hospital, left ventricular ejection fraction, emergent operating status, birth region, educational level, preoperative atrial fibrillation, preoperative cancer, preoperative chronic obstructive pulmonary disease, preoperative diabetes, previous endocarditis, preoperative heart failure, preoperative hypertension, preoperative hepatic disease, preoperative peripheral vascular disease, preoperative stroke, preoperative percutaneous coronary intervention, marital status, valve prosthesis model, disposable family income, and valve prosthesis size.

Mortality

The final model for mortality used a natural spline with three degrees of freedom to model the baseline hazard. Age was included as a natural spline with four degrees of freedom. Body mass index, year of surgery, and estimated glomerular filtration rate were included as natural splines with three degrees of freedom. The other included covariates were patient sex, operating hospital, left ventricular ejection fraction, emergent operating status, concomitant coronary artery bypass grafting, concomitant ascending aortic surgery, birth region, educational level, disposable family income, preoperative atrial fibrillation, preoperative alcohol dependency, preoperative myocardial infarction, preoperative cancer, preoperative chronic obstructive pulmonary disease, preoperative diabetes, previous endocarditis, preoperative heart failure, preoperative hyperlipidemia, preoperative hypertension, preoperative hepatic disease, preoperative peripheral vascular disease, preoperative stroke, preoperative major bleeding event, preoperative pacemaker, preoperative percutaneous coronary intervention, marital status, valve prosthesis size, valve prosthesis model, and preoperative drug abuse.

Table S1. Frequency of the most common valve prosthesis models per xenograft group

Xenograft	Model group	N
Bovine	Perimount	13,276
Bovine	Mitroflow/Crown	1,977
Bovine	Soprano	982
Bovine	Other	368
Porcine	Biocor/Epic	2,612
Porcine	Hancock/Mosaic	1,628
Porcine	Other	179

Table S2. Incidence rates per 100 person years during different follow-up intervals of endocarditis and all-cause mortality following bioprosthetic aortic valve replacement in Sweden between 1997 and 2018

Xenograft	Follow-up time	Endocarditis	All-cause mortality	Late endocarditis
Crude				
Bovine	Overall	0.75 (0.69-0.81)	6.8 (6.6-6.9)	0.59 (0.55-0.65)
Bovine	0 to 1 years	0.94 (0.79-1.11)	6.3 (5.9-6.7)	0.00 (0.00-0.00)
Bovine	1 to 5 years	0.71 (0.63-0.79)	4.0 (3.8-4.2)	0.71 (0.63-0.79)
Bovine	5 to 10 years	0.73 (0.62-0.84)	8.7 (8.4-9.1)	0.73 (0.62-0.84)
Bovine	10 to 15 years	0.69 (0.49-0.93)	15.8 (14.9-16.7)	0.69 (0.49-0.93)
Porcine	Overall	0.65 (0.57-0.74)	8.4 (8.1-8.7)	0.56 (0.49-0.65)
Porcine	0 to 1 years	0.69 (0.46-0.99)	7.0 (6.2-7.8)	0.00 (0.00-0.09)
Porcine	1 to 5 years	0.61 (0.49-0.75)	4.8 (4.5-5.2)	0.61 (0.49-0.75)
Porcine	5 to 10 years	0.66 (0.51-0.83)	9.8 (9.2-10.3)	0.66 (0.51-0.83)
Porcine	10 to 15 years	0.77 (0.51-1.11)	16.2 (15.0-17.4)	0.77 (0.51-1.11)
Age- and sex-adjusted				
Bovine	Overall	0.76 (0.70-0.82)	7.22 (7.07-7.36)	0.60 (0.55-0.65)
Bovine	0 to 1 years	0.94 (0.80-1.11)	6.51 (6.11-6.94)	0.00 (0.00-0.00)
Bovine	1 to 5 years	0.71 (0.64-0.80)	4.15 (3.98-4.33)	0.71 (0.64-0.80)
Bovine	5 to 10 years	0.72 (0.62-0.83)	9.46 (9.11-9.83)	0.72 (0.62-0.83)
Bovine	10 to 15 years	0.68 (0.50-0.93)	17.10 (16.17-18.09)	0.68 (0.50-0.93)
Porcine	Overall	0.68 (0.59-0.77)	7.99 (7.76-8.23)	0.59 (0.51-0.68)
Porcine	0 to 1 years	0.71 (0.49-1.05)	6.68 (5.92-7.54)	0.00 (0.00-0.00)
Porcine	1 to 5 years	0.62 (0.50-0.77)	4.47 (4.15-4.81)	0.62 (0.50-0.77)
Porcine	5 to 10 years	0.69 (0.54-0.87)	9.05 (8.55-9.57)	0.69 (0.54-0.87)
Porcine	10 to 15 years	0.76 (0.52-1.10)	16.18 (15.05-17.40)	0.76 (0.52-1.10)

Age- and sex-adjusted incidence rates were obtained from a Poisson model. CI = confidence interval.

Figure S1. Number of patients who underwent surgical aortic valve replacement in Sweden between 1997 and 2018 with either a bovine or a porcine valve prosthesis.

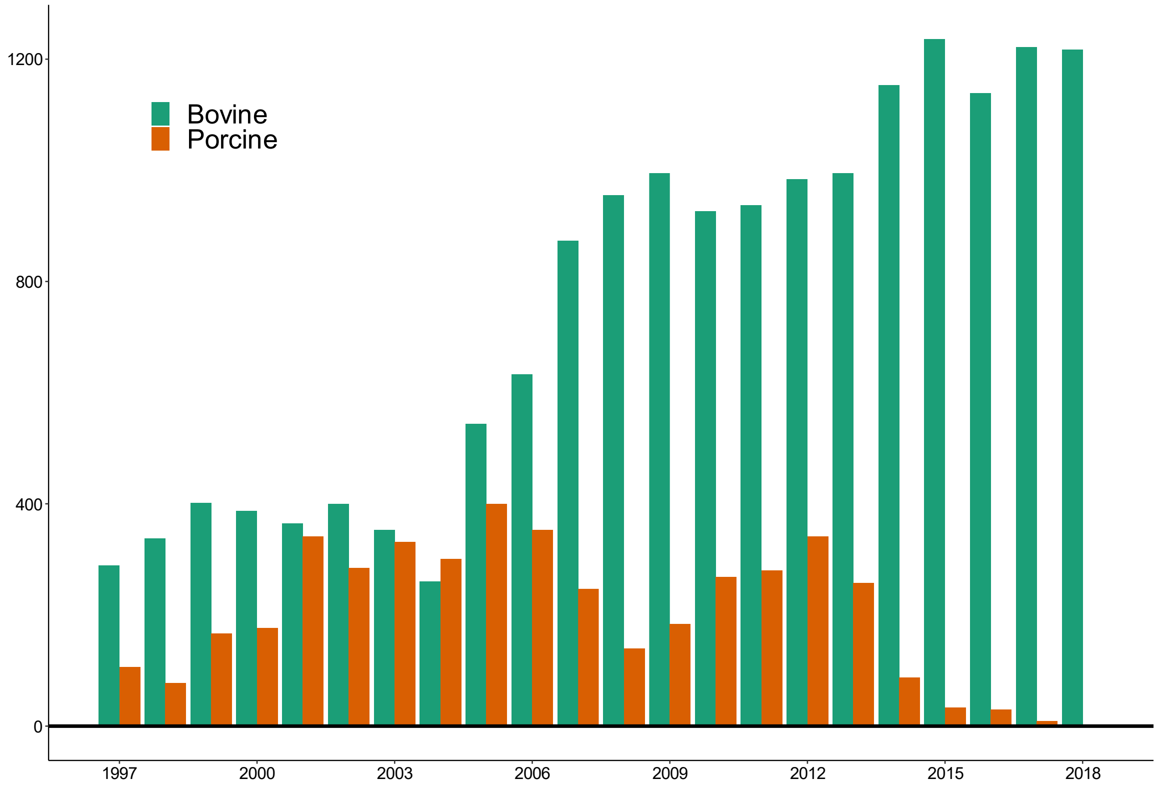

Figure S2. Regression standardized cumulative incidence and difference of late endocarditis in patients who underwent surgical aortic valve replacement in Sweden between 1997 and 2018 and received either a bovine or porcine valve prosthesis.

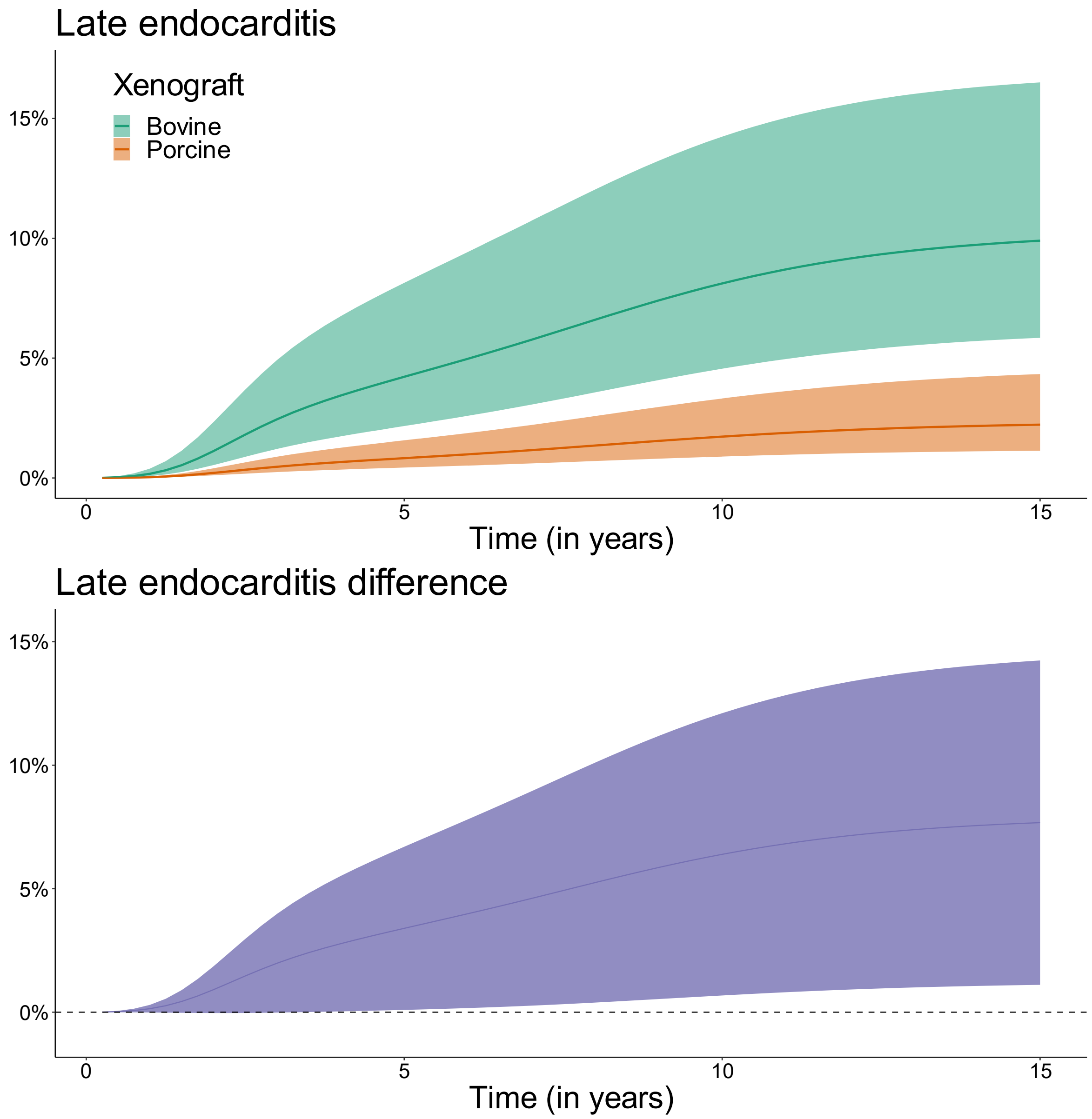

Figure S3. Aalen-Johansen estimated crude cumulative incidence of endocarditis with associated number at risk.

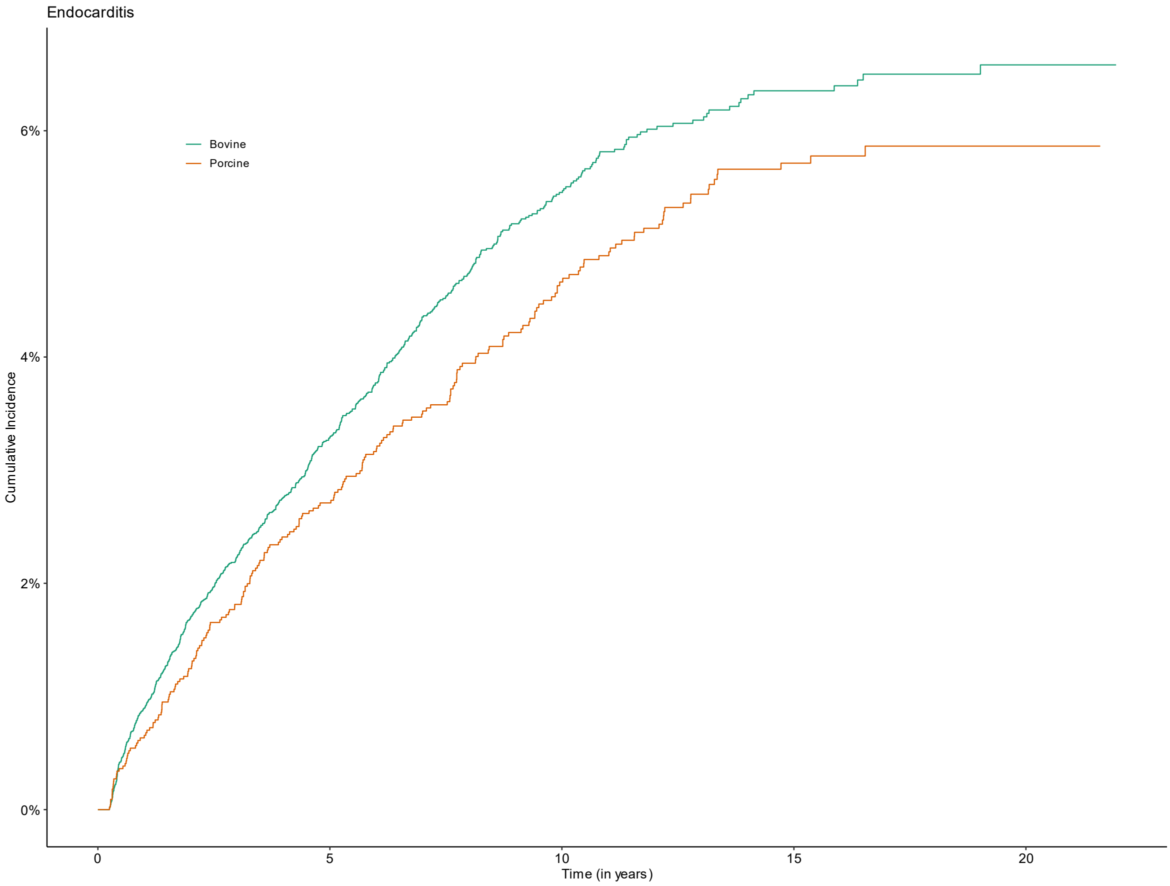

**Bovine**					
At Risk	16603	8092	2600	373	23
Events	0	460	646	686	690
**Porcine**					
At Risk	4419	3202	1286	268	5
Events	0	119	190	218	220

Figure S4. Kaplan-Meier estimated crude survival with associated number at risk table.

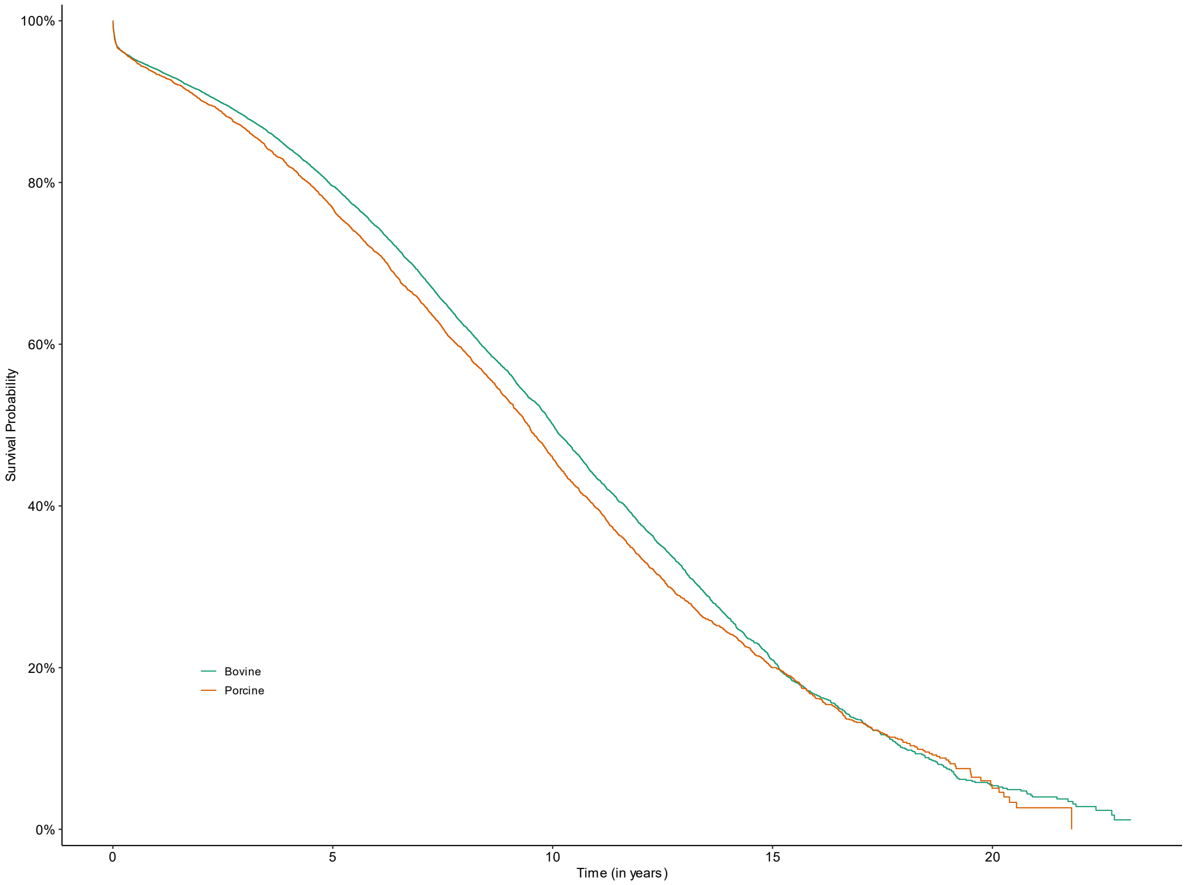

	0	5	10	15	20
**Bovine**					
At Risk	16603	9469	3337	470	39
Events	0	3029	5691	6932	7165
**Porcine**					
At Risk	4419	3344	1439	364	11
Events	0	1021	2201	2898	3048
